# Supplementary material for: TRAIL-induced variation of cell signaling states provides nonheritable resistance to apoptosis
Source: Life Sci Alliance. 2019 Nov 8;2(6):e201900554. doi: 10.26508/lsa.201900554 (PMC6848270; doi:10.26508/lsa.201900554)
Supplement: Supplementary file 1 [file LSA-2019-00554_TableS1.docx]

**Table S1**

| Antibody | Channel | Antibody | Manufacturer | Stock conc | Staining | Used for | Used for |
| --- | --- | --- | --- | --- | --- | --- | --- |
|  | (conjugate) | Clone |  | ug/ml | ug/ml | VISNE | Signaling Diversity |
|  |  |  |  |  |  |  |  |
| pBad (S112) | 141 | 40A9 | CST | 200 | 1 | N | N |
| cCasp3 | 142 | C92-605 | BD | 200 | 1 | N | N |
| p4EBP1 | 143 | 236B4 | CST | 200 | 0.5 | Y | Y |
| RSK2 | 144 | D21B2 | CST | 200 | 0.5 | Y | Y |
| pP38 | 145 | 36/p38 | BD | 100 | 1 | Y | Y |
|  |  |  |  |  |  |  |  |
| pP90RSK | 147 | poly | CST | 200 | 2 | Y | Y |
| pNFkB | 149 | K10-895.12.50 | BD | 200 | 0.5 | Y | Y |
| S6(total) | 150 | 54D2 | CST | 200 | 0.5 | Y | Y |
| pAkt (S473) | 152 | D9E | CST | 100 | 0.5 | Y | Y |
| pMAPKAPK-2 | 153 | 27B7 | CST | 200 | 1 | Y | Y |
|  |  |  |  |  |  |  |  |
| Puromycin | 158 | 12D10 | Millipore | 100 | 1 | N | N |
| pH3 (S28) | 160 | HTA28 | Biolegend | 50 | 0.25 | N | N |
| pBCL-2 (Ser70) | 162 | 5H2 | CST | 200 | 1 | N | N |
|  |  |  |  |  |  |  |  |
|  |  |  |  |  |  |  |  |
| pHSP27 (S82) | 166 | D1H2 | CST | 200 | 0.5 | Y | Y |
| pErk1/2 | 167 | D13 | CST | 200 | 2 | Y | Y |
| Ki-67 | 168 | B56 | BD | 200 | 0.5 | N | Y |
| IkBa | 169 | L35A5 | CST | 200 | 0.5 | Y | Y |
| cParp | 171 | F21-852 | BD | 200 | 1 | N | N |
| pS6 (S235/236) | 172 | N7-548 | BD | 200 | 1 | Y | Y |
| pAMPK | 175 | 40H9 | CST | 200 | 1 | Y | Y |
| MCL-1 | 176 | poly | CST | 200 | 1 | N | N |
|  |  |  |  |  |  |  |  |
